# A Candidate Subspecies Discrimination System Involving a Vomeronasal Receptor Gene with Different Alleles Fixed in *M. m. domesticus* and *M. m. musculus*

**DOI:** 10.1371/journal.pone.0012638

**Published:** 2010-09-09

**Authors:** Robert C. Karn, Janet M. Young, Christina M. Laukaitis

**Affiliations:** 1 Department of Medicine, College of Medicine, University of Arizona, Tucson, Arizona, United States of America; 2 Division of Human Biology, Fred Hutchinson Cancer Research Center, Seattle, Washington, United States of America; National Institute on Aging, United States of America

## Abstract

Assortative mating, a potentially efficient prezygotic reproductive barrier, may prevent loss of genetic potential by avoiding the production of unfit hybrids (i.e., because of hybrid infertility or hybrid breakdown) that occur at regions of secondary contact between incipient species. In the case of the mouse hybrid zone, where two subspecies of *Mus musculus* (*M. m. domesticus* and *M. m. musculus*) meet and exchange genes to a limited extent, assortative mating requires a means of subspecies recognition. We based the work reported here on the hypothesis that, if there is a pheromone sufficiently diverged between *M. m. domesticus* and *M. m. musculus* to mediate subspecies recognition, then that process must also require a specific receptor(s), also sufficiently diverged between the subspecies, to receive the signal and elicit an assortative mating response. We studied the mouse *V1R* genes, which encode a large family of receptors in the vomeronasal organ (VNO), by screening Perlegen SNP data and identified one, *Vmn1r67*, with 24 fixed SNP differences most of which (15/24) are nonsynonymous nucleotide substitutions between *M. m. domesticus* and *M. m. musculus*. We observed substantial linkage disequilibrium (LD) between *Vmn1r67* and *Abpa27*, a mouse salivary androgen-binding protein gene that encodes a proteinaceous pheromone (ABP) capable of mediating assortative mating, perhaps in conjunction with its bound small lipophilic ligand. The LD we observed is likely a case of association rather than residual physical linkage from a very recent selective sweep, because an intervening gene, *Vmn1r71*, shows significant intra(sub)specific polymorphism but no inter(sub)specific divergence in its nucleotide sequence. We discuss alternative explanations of these observations, for example that *Abpa27* and *Vmn1r67* are coevolving as signal and receptor to reinforce subspecies hybridization barriers or that the unusually divergent *Vmn1r67* allele was not a product of fast positive selection, but was derived from an introgressed allele, possibly from *Mus spretus*.

## Introduction

Pheromones, specific substances secreted to the exterior of an organism, communicate information about sex, species and related states to other members of the same species, whereupon the pheromones elicit specific reactions such as behavior and/or endocrine changes [1]. Many pheromones in terrestrial animals are volatile airborne molecules, however, large non-volatile molecules such as peptides and proteins may also be utilized for communication (reviewed in [2], [3]). Olfactory cues represent the primary means of communication in nocturnal animals such as the house mouse [4], [5] and two families of receptors in the vomeronasal organ (VNO), the V1Rs and the V2Rs, are thought to detect pheromonal signals [6]. Studies of putative mouse pheromones have proliferated over the past several decades, however, it is one thing to propose a pheromonal function but quite another to elucidate a mechanism, including identification of the receptor by which the pheromone is recognized. In the case of putative mouse pheromones, receptor identification has generally relied on cell biology experiments which have indicated that the VNO and the accessory olfactory bulb comprise the system receiving and processing the information [7], [8], but in those experiments identification of the specific VNO receptors has been elusive. In this report, we demonstrate a purely genetic and evolutionary approach with which to identify specific receptors for a particular pheromonal function, subspecies recognition [9]–[11], thought to mediate assortative mating where two different subspecies make secondary contact, e.g. the European house mouse hybrid zone described below.

The house mouse, *Mus musculus*, comprises at least three relatively distinct parapatric gene pools given subspecies status by some and full species status by others (for reviews see [12], [13]). Two subspecies, *Mus musculus domesticus* and *M. m. musculus*, occupy distinct geographic ranges in western and eastern Europe, respectively. Where these make contact across southern Danish Jutland and through Central Europe from the Baltic Sea to the Black Sea coast, they form a narrow hybrid zone, which is a region of limited gene exchange [12]–[14]. Two lines of indirect evidence suggest that selection is acting against hybrids: (1) hybrid male sterility and partial female sterility have been described in different crosses of laboratory or wild populations [15]–[23]; and (2) limited introgression of sex chromosome markers as compared to autosomes has been shown across four studied hybrid zone transects [24]–[31]. The reduced fitness of hybrid animals within the zone has been proposed to create a genetic sink, where genes entering the zone are eliminated by selection [32].

Assortative mating is a potentially efficient prezygotic reproductive barrier, which may prevent loss of genetic potential into unfit hybrids [33]–[40]. When partial postzygotic isolation acts in the presence of divergent specific mate recognition systems, selection for increased mating specificity, the phenomenon of reinforcement, may lead to complete speciation [34], [41]–[43]. This idea predicts that if hybrids are less fit, reinforcement should then amplify homo(sub)specific preference most close to a contact zone, a phenomenon called reproductive character displacement. Reinforcement is best studied in closely related or recently divergent taxa, such as the subspecies of house mice, where limited hybridization still occurs and speciation may be incipient. Here, selection may act to reinforce prezygotic isolation in regions of secondary contact, e.g. the European mouse hybrid zone, leading to avoidance of disadvantageous hetero(sub)specific mating. A divergent subspecific mate recognition system, upon which reinforcement in the mouse European hybrid zone is predicated, requires some means by which members of a subspecies can recognize their own subspecies from a foreign one.

Subspecies recognition was originally suggested by studies of polymorphism of a mouse androgen-binding protein gene (*Abpa*, now *Abpa27*) [44] in which different alleles were observed to be fixed in different subspecies [45], [46]. Those observations led to the development of congenic strains that differ only in their *Abpa27* alleles. Subsequent studies of mate recognition involving saliva targets from the congenic strains showed that mice are capable of recognizing their own subspecies from another and choose to mate with their own more frequently than with a foreign subspecies [9]–[11]. Thus one pheromonal function in house mice is recognition of subspecies identity for the purpose of mediating assortative mating and it has been proposed that the mouse VNO is the tissue that recognizes such pheromones [10], [11]. Since that work, evidence has been obtained suggesting that mate preference across the European mouse hybrid zone is a case of reproductive character displacement [9]. This has been observed as increased interest in congenic saliva targets in populations offset from the center of the hybrid zone [9] as predicted by the theory of reinforcement [42], [47], [48]. Recently, Vošlajerová Bímová et al [49] have tested mice across a transect of the mouse hybrid zone for their preferences for saliva from the *Abp* congenic strains and have shown that the incorporation of a reinforcement parameter into the model for the behavioural data creates a significantly better fit than other cline models.

We based the work reported here on the hypothesis that, if there are pheromonal signals mediating subspecies recognition between *M. m. domesticus* and *M. m. musculus*, then there must also be specific receptors that are sufficiently diverged between the subspecies to receive the signal and to elicit an assortative mating response. We chose to study the V1R receptor genes because of their relatively simple structure; they are intron-less genes less than 1 kb in length, whereas V2R genes have many exons spread over ∼20 kb of DNA. We hypothesized that there should be at least one V1R receptor gene exhibiting a high level of divergence, in the form of fixed nonsynonymous differences between the two subspecies of *Mus musculus*, arising by adaptive evolution to allow the receptor to distinguish subspecies-specific signals. We screened a subset of the most likely candidate V1R genes and found that one, *Vmn1r67*, indeed shows a large number of fixed changes between the two subspecies and an unusual pattern of evolution that may suggest its involvement in subspecies recognition.

## Results

### Screening for *V1R* genes significantly diverged between *M. m. domesticus* and *M. m. musculus*

We began our search for a *V1R* gene(s) with different haplotypes fixed in the two subspecies, *M. m. domesticus* and *M. m. musculus*, by screening the 392 *V1R* genes for those with the most SNP differences between the wild-derived inbred strains WSB and PWD in the Perlegen SNP data [50]. The microarrays used by Perlegen for resequencing were designed from the mm6 version of the mouse genome assembly, which is older and more incomplete than the current build; thus, some regions of the genome simply were not assayed with this technology.

We reasoned that positive selection would maximize the number of fixed differences between the two haplotypes allowing discrimination of signals communicating subspecies identification (although there may be other explanations for large numbers of fixed differences – see Discussion). We ranked the 392 *V1R* genes according to how many SNPs were reported by Perlegen to have different genotypes in WSB and PWD (Table S1). The screen results showed that 165 V1Rs (42%) contain at least one SNP where WSB and PWD are reported to have different genotypes, while the rest (227) had no reported WSB-PWD differences. Excluding pseudogenes, the ten *V1R* genes with the most differences between the two strains were *Vmn1r31* (9), *Vmn1r75* (8), *Vmn1r67* (6), *Vmn1r39* (5), *Vmn1r12* (5), *Vmn1r64* (5), *Vmn1r62* (5), *Vmn1r175* (5), *Vmn1r61* (5), and *Vmn1r37* (4); the number of SNPs where WSB and PWD have different alleles appears in parentheses for each gene. To determine how many SNPs in these candidate V1R genes might represent fixed nucleotide differences between the two subspecies of interest, we produced DNA sequences of the ten top-ranked genes from the Perlegen screen in at least two wild-derived inbred strains of each subspecies (except *Vmn1r61*, where only a single SNP of any kind was evident between the single *M. m. domesticus* and *M. m. musculus* strains we sequenced). Only *Vmn1r67* (also known as V1RE10) had a substantial number of fixed differences (24 differences) between the sets of strains representing the two subspecies. We did not pursue the other nine candidate genes because most had no fixed differences between the subspecies (only *Vmn1r12* had any, with two in 906 bp; File S1). Therefore, most allelic differences reported between WSB and PWD represent polymorphisms in populations of both subspecies.

**Figure 1:**
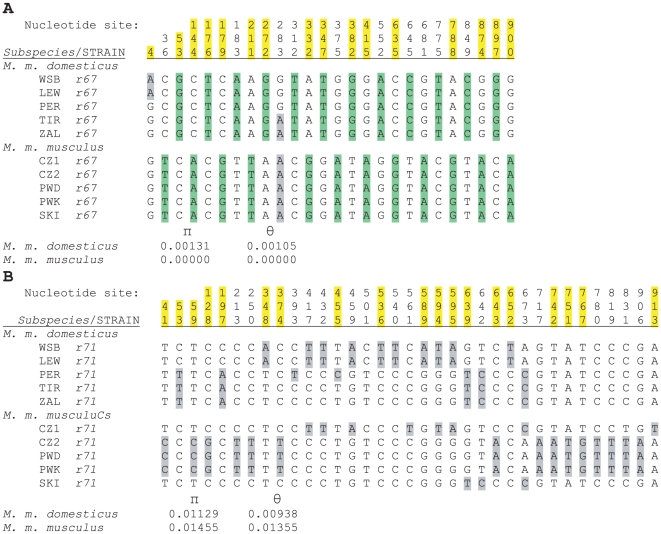
A comparison of polymorphism and divergence in two *V1R* genes. Reduced data sets summarizing SNP differences for *Vmn1r67* (Panel A) and the closely linked gene *Vmn1r71* (Panel B) in five wild-derived inbred strains each for *M. m. domesticus* and *M. m. musculus*. Strain abbreviations are: LEW = LEWES; PER = PERA; TIR = TIRANO; ZAL = ZALENDE; CZ1 = CZECHI; CZ2 = CZECHII; and SKI = SKIVE. Fixed differences (divergence) between *M. m. domesticus* and *M. m. musculus* are highlighted in green (there are 24 in *Vmn1r67* and none in *Vmn1r71*) and polymorphisms in both genes in the two subspecies are highlighted in gray to facilitate comparison. In addition, nonsynonymous sites in *Vmn1r67* are indicated by yellow highlighting of the site numbers. Nucleotide diversity (π) and nucleotide polymorphism (θ) were calculated for both subspecies using DNAsp and are shown for each dataset. Full sequence data appear in Files S2 and S3.

We sequenced the *Vmn1r67* gene (915 bp) in three more wild-derived inbred strains of each subspecies to bring the total number of sequences to five each (File S2). For comparison, we also sequenced *Vmn1r71* (921 bp), also known as V1RE13 (File S3), one of the *V1R* genes for which Perlegen did not report any differences between WSB and PWD (Table S1). Figure 1 shows the SNPs found in both *V1R* genes in these ten strains. Of 26 total variable nucleotide sites in *Vmn1r67*, 24 are fixed differences representing divergence in *Vmn1r67* between the two subspecies, while only two are polymorphisms, both of which are in the *M. m. domesticus* subspecies. Of the 24 fixed sites in *Vmn1r67*, 15 are nonsynonymous (i.e., result in amino acid substitutions) while nine are synonymous. Of 35 total variable nucleotide sites in *Vmn1r71*, we found no fixed differences (i.e., no divergent sites) between the two subspecies; all differences were polymorphisms in one or both strains. As expected, these extreme differences in divergence and polymorphism between the two *V1R* genes are reflected in much higher (∼ten-fold) nucleotide diversity (π) and nucleotide polymorphism (θ) in *Vmn1r71* compared to *Vmn1r67* (Fig. 1). Nucleotide diversity and nucleotide polymorphism had previously been reported to be 0 for *Abpa27* in five *M. m. domesticus* samples from widely separated geographical locations [51].

Perlegen reported 19 SNPs for *Vmn1r67*, at only six of which did they make a genotype assignment (G, A, T or C) for both WSB and PWD. At the other thirteen sites, one strain genotype was reported only as “N” (ambiguous site). By contrast, we discovered 26 SNPs by sequencing the DNA of WSB and PWD. Thus, we confirmed polymorphisms at all of the sites reported by Perlegen, and confirmed the genotypes called for each strain at 18 of 19 SNP positions. Only one Perlegen genotype disagreed with our sequencing data (Perlegen: G; our data: C; PWD genotype at nucleotide 897 in Fig. 1). According to our sequencing, this is a fixed SNP difference between the two subspecies, not a polymorphic site. Thus the Perlegen data ascertained 73% of the SNP sites but reported high quality genotypes that differ between WSB and PWD at only 23% of those sites, instead providing ambiguous calls at many sites. We discovered seven additional sites that Perlegen did not report as SNPs.

### Evolution of *Vmn1r67*

**Figure 2:**
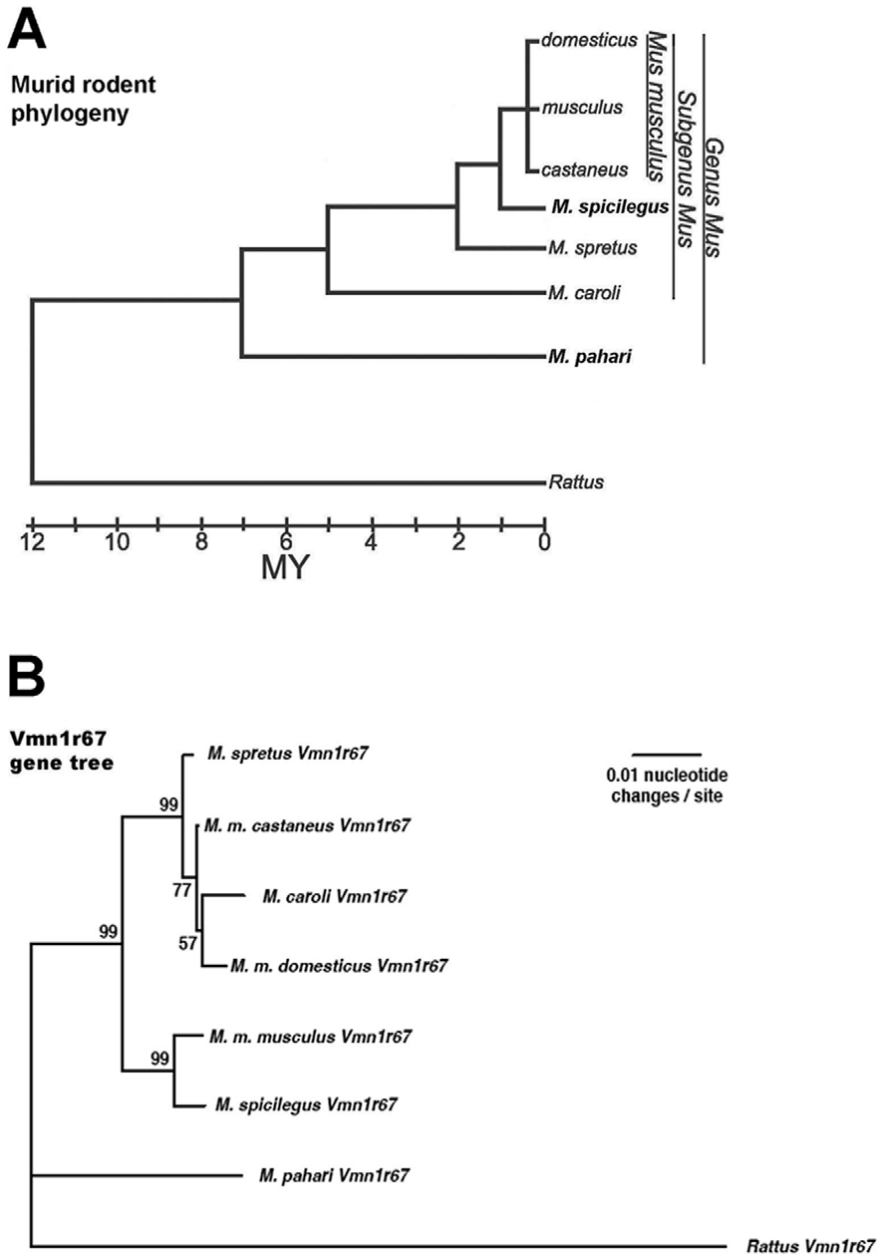
A comparison of a murid rodent phylogeny and a *V1R* gene phylogeny. Panel A: A canonical phylogeny of murid rodents (adapted from [57]); Panel B: A *Vmn1r67* gene phylogeny for murid rodents using F84 nucleotide distances and neighbor-joining, with bootstrap values expressed as percentages (see Methods). Full sequence data appear in File S4.

A possible interpretation of the high divergence between the *Vmn1r67* alleles fixed in *M. m. domesticus* and *M. m. musculus*, combined with low/absent polymorphism is that positive selection has acted on this gene in the house mouse (see Discussion for an alternative explanation). In order to assess this, we used maximum likelihood methods employed in the CODEML program of the PAML package [52]–[54]. We obtained *Vmn1r67* amplicons from the third *Mus musculus* subspecies (*M. m. castaneus*) and four other species of the genus *Mus: M. spicilegus, M. spretus*, *M. caroli*, *M. pahari* with polymerase chain reaction (PCR) and sequenced them. Two possible *Rattus norvegicus Vmn1r67* orthologs were obtained from [55]. Orthology of all sequenced *Vmn1r67* genes from taxa of the genus *Mus* was confirmed by Blast searches against the mouse genome [56]. The phylogeny of Chevret et al. [57] was used for the rodent species (Fig. 2A) for the PAML tests and the three subspecies of *M. musculus* were treated as an unresolved polytomy. See File S4 for the *Vmn1r67* sequence data used in CODEML analysis. *Vmn1r67* showed significant signs of positive selection within murid rodents when the species phylogeny was used as the CODEML guide tree. However, we also noticed that the evolution of the *Vmn1r67* sequences appears not to follow the species tree (Figure 2, File S4), and that the sequence data support an alternate tree (Figure 2B) much better than the species tree. This unexpected finding may be due to phenomena including one or more introgression events, incomplete lineage sorting, and/or homoplasies (recurrent mutations). Such introgression or homoplastic mutations might even have been fixed due to positive selection.

PAML's author, Ziheng Yang, has suggested that the gene tree should be used when it differs substantially from the species tree (http://www.ucl.ac.uk/discussions/viewtopic.php?t=7850). When the same taxa were analyzed with CODEML using the *Vmn1r67* gene phylogeny as the guide tree (Fig. 2B), we obtained nonsignificant results (not shown), evidently because the *Vmn1r67* gene phylogeny is widely incongruent with the murid rodent species phylogeny. The branch-sites model [58] also failed to yield significant evidence of positive selection using the gene tree. We reconstructed ancestral sequences using CODEML and parsed the rst output to look for evidence of homoplasy that could explain the substantial incongruence between the two phylogenies. Using the species tree, we encountered 19 apparent homoplasies but none when we used the gene tree (File S5). Most of the fixed nonsynonymous SNP differences (14/15) we observed between the *Vmn1r67* genes of the two *M. musculus* subspecies are at sites exhibiting homoplasy if the species tree is assumed. It is not clear whether these changes indeed represent true recurrent mutations or whether this portion of the *Mus* genome has evolved with a very different history than the genomes in which it resides.

We used the Phyre threading program on one of the *M. m. domesticus* sequences (PERA) to identify the most likely structural model, d1ln6a (Family A G protein-coupled receptor-like; rhodopsin-like) with 100% confidence mapping of the 305 amino acids of Vmn1r67 onto 309 total positions of the model with an identity of 10%. Fifteen Vmn1r67 amino acid sites were not captured by the threading program: seven in the N-terminal sequence, four between sites 115 and 120, two between sites 157 and 160, and two at the C-terminus of the model. The d1ln6a model is a seven transmembrane domain structure consistent with the general structure for V1R receptor proteins and we used it to produce a diagram of the Vmn1r67 protein that reflects the details of the transmembrane helix and coil structures.

**Figure 3:**
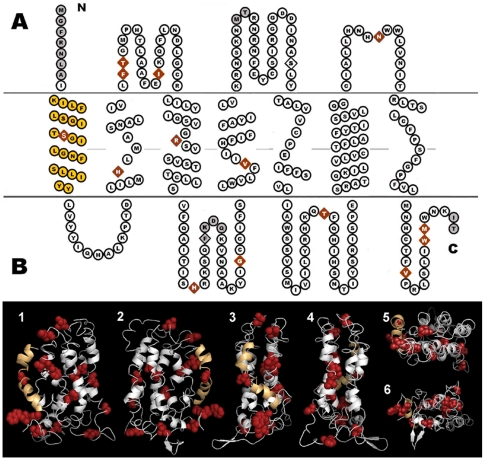
Amino acid differences in a V1R gene between *M. m. domesticus* and *M. m. musculus*. Diagrammatic representation of the amino acid sequence of Vmn1r67 (Panel A) and the three-dimensional structure on which it was threaded (Panel B); amino acids in Panel A that were not threaded on the d1ln6a model in Panel B are shaded gray. The amino acid positions affected by fifteen nonsynonymous differences fixed between *M. m. domesticus* and *M. m. musculus* (File S6) are shown as diamonds. Fourteen nonsynonymous sites detected by a test for homoplasy (File S5) are colored red. For orientation to the three-dimensional models in Panel B, the first helix is colored light orange. Panel B views are 1) front, 2) rear, 3) left side, 4) right side, 5) top and 6) bottom.

Figure 3A is a diagram of the amino acid sequence of Vmn1r67 with the fixed amino acid differences between *M. m. domesticus* and *M. m. musculus* represented with diamond shapes (amino acid sequences appear in File S6). Amino acid residues affected by nonsynonymous sites where apparent homoplasy was detected assuming the species tree are mapped onto the sequence in red. The figure also shows the same sites mapped in red onto three-dimensional representations of the structure (Fig. 3B). The most important conclusion from this comparison is that the majority of amino acid residues detected with the test for apparent homoplasies are also diverged between *M. m. domesticus* and *M. m. musculus* Vmn1r67 receptors. Most of the amino acid residues that differ between the two subspecies due to fixed SNPs appear in either extracellular loops (four) or intracellular loops (six), although four appear in transmembrane helices (see Discussion).

### Linkage disequilibrium (LD) between *Vmn1r67* and *Abpa27*

**Figure 4:**
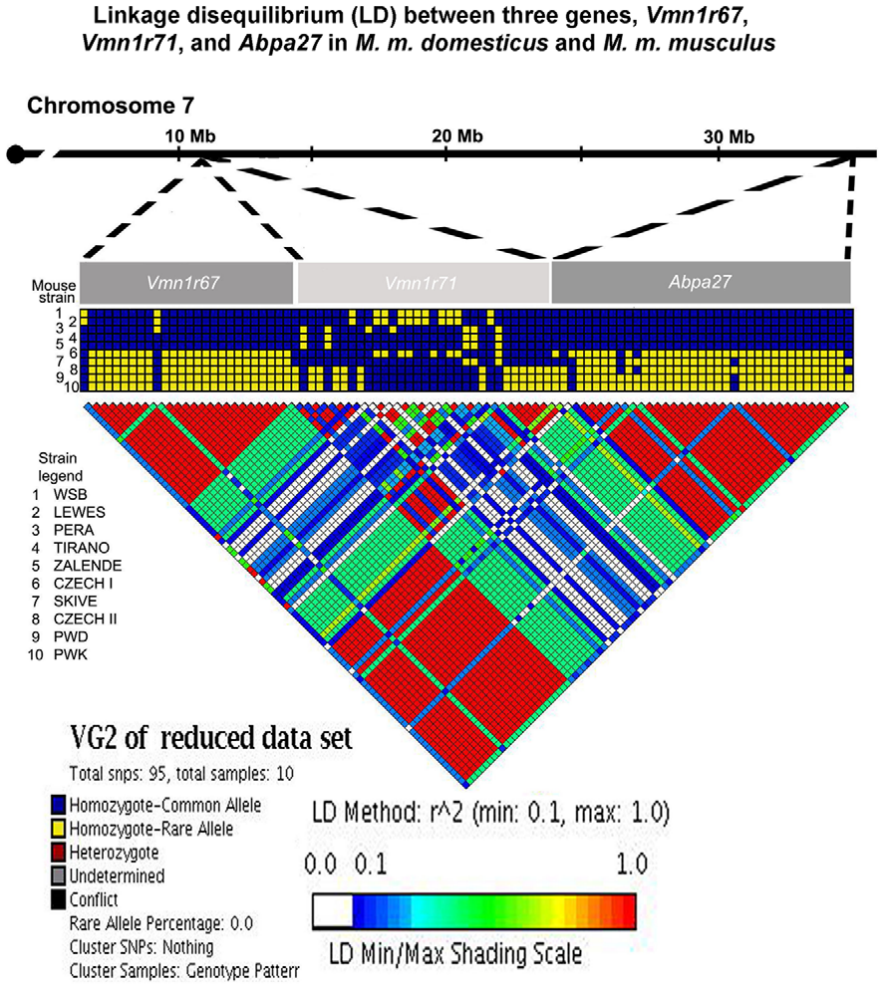
Linkage disequilibrium (LD) between *Vmn1r67* and *Abpa27*, with the gene *Vmn1r71* mapping between them. The proximal end of mouse chromosome 7 shown at the top of the figure represents the relative positions of the three genes which appear diagrammed as gray-shaded blocks below the chromosome and over their portion of the LD diagram. A comparison of the degree of LD, represented as r^2^ values, appears in the triangle at the bottom of the figure and a shading scale for LD minimum to maximum appears under the LD diagram. The wild-derived inbred mouse strains used in this study are numbered on the left side of the LD diagram with a strain legend immediately below. The genes *Vmn1r67* and *Abpa27* show strong LD, while *Vmn1r71* shows no significant LD with either of them.

In light of the data we present here, suggesting that *Vmn1r67* has alleles fixed in *M. m. domesticus* and *M. m. musculus*, and the previously reported fixed alleles for *Abpa27* in the three subspecies of the house mouse [59], [60], we evaluated linkage disequilibrium (LD) between these two genes. In order to test whether LD might be due to a very recent selective sweep covering the ∼23 Mb between them, we included *Vmn1r71*, which lies between the two genes. Sequencing *Abpa27* (1185 bp) in the same ten wild-derived inbred strains in which we sequenced *Vmn1r67* and *Vmn1r71* produced the sequences predicted from previous studies [46], [51], [61], [62]. Figure 4 illustrates LD for the reduced data set of the three genes. Clearly there is strong LD between *Vmn1r67* and *Abpa27* (red regions indicating maximum r^2^ in the data) but not between *Vmn1r71* and either of those two. These observations indicate a strong association between *Vmn1r67* and *Abpa27*, and the lack of LD with *Vmn1r71* argues against residual physical linkage resulting from a very recent selective sweep across the entire *Vmn1r67-Abpa27* region. This is not surprising given that the distance between the *Vmn1r67* and *Abpa27* genes (23 Mb) is substantially larger than that expected for strong linkage disequilibrium (LD), which occurs over a distance that varies depending on the genomic region, but is usually on the order of tens of kilobases [63].

## Discussion

### The origin of the prediction of vomeronasal receptors differentiating the *Mus musculus* subspecies

We predicted that, if *M. m. domesticus* and *M. m. musculus* are each capable of distinguishing members of their own subspecies from members of another subspecies, there should be at least one vomeronasal receptor that recognizes a distinct difference, i.e. a signal or signals that advertise subspecies identity. In order to accomplish this recognition function there should be at least one V1R receptor encoded by a gene exhibiting a high level of divergence, in the form of fixed nonsynonymous changes between the two subspecies arising by adaptive evolution. Although our initial hypothesis was neutral with regard to the pheromone that might signal the subspecies difference, only one mouse pheromone system, salivary androgen-binding protein (ABP) has been shown to provide a recognizable cue of subspecies status between the subspecies that form the mouse hybrid zone [9]–[11], [49]. Even though V1R receptors are not normally thought to recognize proteinaceous pheromones, ABP appears at this time to be the best candidate pheromone system for mediating prezygotic mating isolation [64] leading to subspeciation, whether through the protein structure itself or through the lipophilic ligand it binds.

### The bioinformatic screen for subspecies-recognition receptor gene candidates

Since the total collection of *V1R* receptor genes numbers 392, we reasoned that we should begin our effort to find a highly diverged *V1R* by selecting a subset of those with the highest number of fixed differences between the wild-derived strains representing the two subspecies in Perlegen SNP data. To that end, we designed the screen we described above. Out of the top ten *V1R* genes returned by our bioinformatic screen of Perlegen SNP data, only one, *Vmn1r67*, had a significant number of fixed SNP differences (24/26), revealed by DNA sequencing in five wild-derived strains for each of the two subspecies *M. m. domesticus* and *M. m. musculus*. In most other cases, WSB-PWD SNPs represented polymorphisms that segregate within both subspecies, rather than fixed differences. While this suggests that our screening approach to identifying candidate subspecies-recognition receptor genes is naïve and somewhat inefficient (∼10%), it allowed us to obtain one such gene rapidly and inexpensively. We note that more than half (58%) of the 392 *V1R* genes had no WSB-PWD differences reported by Perlegen. This clearly reflects the conservative nature of Perlegen data because our DNA sequencing revealed 35 SNP differences in *Vmn1r71*, a gene reported as having no SNP differences. Therefore, our screen remains incomplete, but provided one excellent candidate for a V1R gene involved in subspecies recognition.

### Evolution of *Vmn1r67*

The number of fixed SNP differences in *Vmn1r67* suggests that this gene diverged dramatically between the two subspecies accompanied by the acquisition of very little polymorphism (only two sites in *M. m. domesticus*). By contrast, *Vmn1r71* had 35 SNP differences and all of those were polymorphisms within one or both subspecies. Returning to *Vmn1r67*, nearly two-thirds of the fixed SNP differences (15/24) were nonsynonymous substitutions. While CODEML did not predict positive selection using the gene phylogeny as the guide tree, our observations of low polymorphism and high divergence are suggestive of selection for divergence in this region between the *M. m. domesticus* and *M. m. musculus* subspecies. The extreme incongruence we observe between the gene tree and species tree is intriguing, and may be due to some combination of introgression (see below), incomplete lineage sorting, and/or fixation of recurrent mutations, perhaps under the influence of positive selection to fix divergent alleles. We suggest that *Vmn1r67*'s unusual evolution in *M. m domesticus* and *M. m. musculus* may have occurred in response to a parallel evolution of unique *Abpa27* signals in the two subspecies, explaining the strong LD between *Vmn1r67* and *Abpa27*. Thus, it appears that the LD we report here is a case of association between *Vmn1r67* and *Abpa27*, rather than residual physical linkage from a very recent selective sweep, because an intervening gene, *Vmn1r71*, shows significant polymorphism within subspecies but no fixed divergence between the two subspecies. In advancing the view that the LD we have observed may be explained by functional association, we also note that there may be many such regions in the genome that are mutually fixed between the subspecies. However, in conceiving our experimental approach, we purposely narrowed the field substantially by examining a group of vomeronasal receptors some of which could be candidates for ABP receptor(s) and association between *Vmn1r67* and *Abpa27* is at least a plausible explanation. We also note that among nine other V1Rs for which we obtained sequences, there were only two nucleotide positions showing fixed differences between the two subspecies (File S1), establishing *Vmn1r67*'s pattern of divergence as being unusual.

The hypothesis that rapid positive selection produced the unusual divergence of the *Vmn1r67* alleles in *M. m. domesticus* and *M. m. musculus* is not the only possible explanation of the observations we report here. An alternative explanation is that the *M. m. domesticus* allele was derived from an introgressed allele, for example from *M. spretus* or *M. macedonicus*. In fact, *M. m. domesticus* is broadly sympatric with *M. spretus* both on the southern shore and the northern shore of the Mediterranean Sea, as well as with *M. macedonicus* in the Middle East. Alternatively or in addition, the *M. m. musculus* allele could have been derived from an allele introgressed from another species of *Mus* because it is sympatric with *M. spicilegus* in Central Europe (see [12] for a review of the distributions of these taxa of the genus *Mus*). There is precedence for such an introgression pattern [65] and so we explored this by comparing the sequences of *Vmn1r67* alleles from *M. spretus*, *M. macedonicus* and *M. spicilegus*, as well as with the allele in the *M. m. castaneus* subspecies (File S4). This comparison revealed that the *Vmn1r67* allele in each differs from those in the two subspecies of *M. musculus* we studied here. Thus we conclude that, while we cannot rule out the possibility that either *M. m. domesticus* or *M. m. musculus*, or both acquired a *Vmn1r67* allele from other *Mus* species, we can at least rule out very recent acquisition from these taxa that are known to be sympatric with one or the other of them.

### Coevolution of *Vmn1r67* and *Abpa27*

ABP is the only one of the three proteinaceous pheromone families (ABPs, ESPs and MUPs) that has been shown to have a different allele fixed in each of the three *Mus musculus* subspecies (*Abpa27^a^* in *M. m. domesticus*, *Abpa27^b^* in *M. m. musculus* and *Abpa27^c^* in *M. m. castaneus*; [45], [46]; see [44] for revised nomenclature). Because ESPs have been discovered only recently [66], [67], it is not yet known whether any of them show fixed differences between rodent taxa. Fixed differences in MUPs between the *Mus musculus* subspecies have been sought extensively by others, but not found. Indeed, both ESPs and MUPs have been hypothesized to mediate recognition of individual characteristics including gender [68], rather than recognition of subspecies. MUPs may even allow detection of predators [69]. *Abpa27* is also the only proteinaceous pheromone for which there is population genetic evidence that the different fixed alleles came about by positive selection [51], [61]. Moreover, Karn and Laukaitis [70] previously reported evidence for substantial recent gene duplication and copy-number variation in the *Abp* region, especially in mouse strains with all or part of the *M. m. musculus* or *M. m. castaneus Abp* gene region. By contrast, they did not find evidence for any particularly remarkable recent duplication or copy-number variation in the other two pheromone gene families (ESPs and MUPs). They concluded that there is a striking similarity between the volatility for copy-number variation of *Abp* genes and that reported by others for chemosensory receptor genes, especially the *V1R* genes [71], [72] and speculated that some of the *V1R* genes encode vomeronasal receptors recognizing ABP molecules, which are coevolving to provide a recognition system to reinforce subspecies and species hybridization barriers. At this time we can only suggest that the data we report here are consistent with the idea that *Abpa27* and *Vmn1r67* are coevolving as signal and receptor, respectively. Nonetheless this idea is entirely consistent with the evolution of a pheromonal system resulting in incipient reinforcement acting on behavioural isolation traits in the European mouse hybrid zone and our future research will focus on directly testing it.

### Structural analysis

Most of the sites showing fixed differences representing divergence at nonsynonymous nucleotides between the two subspecies affect amino acid residues found in either extracellular loops (four) or intracellular loops (seven), with only four in transmembrane helices. These nonsynonymous sites affect eleven amino acid residues found in either extracellular or intracellular loops (Fig. 3). The sites in the extracellular loops are candidates for interaction with the ABP dimer, which has all of its selected sites on one exterior face [73]. It is not entirely clear what the function of the seven sites in intracellular loops and the four in the transmembrane helices is. It may be that a clue to the function of these residues lies in ABP's ability to bind male sex steroids and/or other ligands, possibly as a means of enhancing the signal interaction with the receptor. While laboratory studies have shown that ABP is capable of binding male sex steroid hormones [74], [75], the identity of the lipophilic ligand actually bound by ABP in nature has not been identified, and its potential role in ABP's function is not understood. However, it has been shown that mouse saliva contains approximately equal quantities of two different ABP dimers, one composed of the alpha subunit (the subunit encoded by *Abpa27*) disulfide-bridged to the beta (the subunit encoded by *Abpbg27* – see [44] for current nomenclature) and the other of the alpha subunit disulfide-bridged to the gamma subunit (the subunit encoded by *Abpbg26;* [59]). Expression studies have shown that other *Abpa* and *Abpbg* genes are expressed in various tissues but dimeric combinations involving them have yet to be described [76]. Karn and Clements [77] studied the two dimers in saliva (see above) and showed that they bind testosterone and dihydrotestosterone (DHT) with different affinities. If ligand binding provides a mechanism for either dimer to impart information about subspecies status or to enhance ABP's ability to communicate subspecies status (analogous to turning up the volume of a radio), this may explain why we found fixed nonsynonymous sites in transmembrane helices and in intracellular loops, where a lipophilic ligand might be found, as well as in extracellular loops, where an environmental pheromonal protein would bind.

### Physical vs. genetic evidence for pheromone receptors

A number of studies have identified the mouse VNO as the tissue containing receptors for pheromones (reviewed in [7], [8]). Here we have used a genetic approach to look for a candidate receptor(s) for a putative subspecies recognition pheromone, ABP. Admittedly the approach is an indirect one but it has the efficacy of identifying candidate receptor genes that can then be subjected to other studies, including experiments with mouse strains in which either the signal gene (e.g., *Abpa27*) and/or the receptor gene (in this case, *Vmn1r67*) has been knocked out. Additional or alternative experiments involving mate-choice behavior, using subject mice with recombinant *Vmn1r67*- *Abpa27* haplotypes may not only reinforce the conclusion that the Vmn1r67 receptor recognizes the ABP signal but may also reveal more details about the interaction of the receptor and the signal. In any event, future work should also involve direct electrophysiological experiments that will corroborate the genetic identification of a VNO receptor for the ABP molecules proposed to advertise subspecies identity in house mice, and possibly species identity in other rodents of the genus *Mus*.

## Materials and Methods

### Materials

Genomic DNA from wild-derived inbred *Mus musculus domesticus* strains WSB/EiJ, LEWES/EiJ, PERA/EiJ, TIRANO/EiJ, ZALENDE/EiJ; wild-derived *M. m. musculus* strains CZECHI/EiJ, CZECHII/EiJ, PWD/PhJ, PWK/PhJ and SKIVE/EiJ; and wild-derived *M. m. castaneus* (CAST/EiJ), *M. spicilegus* (PANCEVO/EiJ), *M. spretus* (SPRET/EiJ), *M. caroli* (CAROLI/EiJ) and *M. pahari*/EiJ were obtained from Jackson Laboratory. The *M. macedonicus* sample was trapped in Iran by Pavel Munclinger. PCR and DNA sequencing primers were obtained from Bioneer; primer sequences and conditions are available from the authors upon request.

### Screening for maximum SNP differences between *M. m. musculus* and *M. m. domesticus*

We downloaded Perlegen's mouse SNP genotype data [50] (release 4, where SNP coordinates are given relative to Build 37 of the mouse genome assembly, mm9) from http://mouse.perlegen.com. These data were obtained by using an array “re-sequencing” method for key inbred and wild-derived mouse strains. Other strains' genotypes were inferred based on homology blocks determined by similarity of tag SNPs in those strains to the re-sequenced strains. PWD and WSB were both directly re-sequenced and their genotypes were not inferred. Perlegen data is intended to be conservative and a false negative rate of 67% has been estimated [78] and we note that, because the Perlegen data underestimate both SNP numbers and number of allelic differences between the two strains analyzed, our V1R screen remains incomplete. Using a custom Perl script, we determined whether PWD and WSB had different genotypes for each SNP (conservatively not counting “N” genotypes as differences). The same Perl script then determined for each *V1R* gene the total number of SNPs with genotypes that differ between PWD and WSB using the mm9 coordinates of the 392 V1Rs found in a previous study [55]. We selected the ten intact V1R genes with the most reported WSB-PWD differences for further study.

### Molecular methods

The top ten *V1R* genes identified with the SNP screen described above were amplified by PCR and sequenced. The PCR products were evaluated on 1% agarose gels and diluted 1∶4, and were sequenced by the UAGC facility at the University of Arizona. The *V1R* sequences obtained from other species were checked against their known mouse genome coordinates with the BLAT tool on the UCSC genome browser [56], [79], [80].

### Data analysis

DNA sequence traces were edited with Chromas 2.3 (http://www.technelysium.com.au). DNA sequence alignment, coding region assembly, and *in silico* translation were done using the DNAsis Max program 2.0 (Hitachi). Nucleotide diversity (π) and nucleotide polymorphism (θ) were calculated for both subspecies using DnaSP 5.10 [81]. Positive selection was assessed in the program CODEML in the PAML 4.4 package [53], [78]). The phylogeny of Chevret et al. [57] was used for the mouse species for initial PAML tests. The three subspecies of *M. musculus* were treated as an unresolved polytomy. We also followed Yang's advice that the gene tree should be used rather than the species tree if the two are incongruent (http://www.ucl.ac.uk/discussions/viewtopic.php?t=7850), which required constructing a *Vmn1r67* gene phylogeny. The dnadist program of the PHYLIP package 3.68 [82] was used to calculate nucleotide distances using the F84 model and a tree was constructed from those distances using the neighbor-joining method [83], as implemented in PHYLIP's neighbor program [82]. PHYLIP's seqboot was used to calculate bootstrap values from 1000 replicate datasets, again using F84 distances with neighbor-joining [82]. We also constructed a maximum likelihood gene tree with PHYLIP's dnaml [84] and obtained the same topology.

Three different comparisons of neutral and selection models were made (M1 vs. M2, M7 vs. M8, and M8A vs. M8 [54], [85], [86]. Model M1 (neutral) allows two classes of codons, one with *dN/dS* over the interval (0,1) and the other with a *dN/dS* value of one. Model M2 (selection) is similar to M1 except that it allows an additional class of codons with a freely estimated *dN/dS* value. Model M7 (neutral) estimates *dN/dS* with a beta-distribution over the interval (0, 1), whereas model M8 (selection) adds parameters to M7 for an additional class of codons with a freely estimated *dN/dS* value. M8A (neutral) is a special case of M8 that fixes the additional codon class at a *dN/dS* value of one. We also conducted “branch-sites” CODEML analysis [58] comparing Model A with the corresponding neutral model. As well as sites under purifying selection and neutrally evolving sites, Model A allows for a class of amino acid sites that only experience positive selection on one or more selected “foreground” branches of the tree, rather than over the entire phylogeny under consideration. Tests were performed specifying as foreground branches either the *M. m. domesticus* terminal branch alone, the *M. m. musculus* terminal branch alone, or both terminal branches. In order to detect apparent homoplasies when the species tree is assumed, we used CODEML to reconstruct ancestral sequences and list nucleotide changes occurring along each branch (with settings model = 1 to allow each branch its own *dN/dS* value, and NSsites = 0 to allow only one category of site). We then used a custom Perl script to parse CODEML's rst output file to look for apparent homoplasies (identical nucleotide changes happening on more than one branch of the tree). No such homoplasies were detected if the gene tree is assumed.

Two possible rat orthologs of *Vmn1r67* were obtained from Young et al [55]. Both are located on chromosome 1 of the Nov. 2004 rat genome assembly, at coordinates 63137741–63138658 and 63198769–63199686 (both on the forward strand). We used the first of these two genes in our codeml analysis, and provide its sequence in File S4. Unlike most *V1R*s, there does not seem to have been extensive post-rat-mouse-speciation duplication in this gene [55]. The three-dimensional structure of Vmn1r67 was modeled using the PHYRE 0.2 threading program (http://www.sbg.bio.ic.ac.uk/phyre/), and the resulting model was visualized using PYMOL (open-source 1.2.8; http://www.pymol.org/). Sites showing fixed *domesticus*-*musculus* changes in *Vmn1r67* were mapped onto structural models using PYMOL and transferred to a figure of the amino sequence using PHOTOSHOP 10.1.

Linkage disequilibrium (LD) was assessed using the linkage disequilibrium viewer of the genome variation server (http://gvs.gs.washington.edu/GVS). The *Vmn1r67, Vmn1r71 and Abpa27* sequences from five strains each of the *M. m. domesticus* and *M. m. musculus* subspecies were concatenated and aligned using BioEdit 7.0.9.0 (6/27/07) [87]. Nucleotide sites varying between the samples were combined into a single reduced data set, manually reformatted into the recommended format and uploaded. The LD image output was saved and a figure constructed with labels in PHOTOSHOP.

## Supporting Information

Table S1 Raw data from the Perlegen screen. Screen of the Perlegen SNP data on *V1R* genes ranked from most PWD-WSB SNPs to least.
(0.05 MB XLS)

File S1 The partial DNA sequences of nine of the top ten *V1R* genes selected in our screen of Perlegen SNPs. These were rejected as having few or no fixed differences between PWD and WSB.
(0.05 MB TXT)

File S2 The five *M. m. domesticus* and five *M. m. musculus Vmn1r67* gene sequences that contributed to the reduced data set shown in Figure 1A.
(0.01 MB TXT)

File S3 The five *M. m. domesticus* and five *M. m. musculus Vmn1r71* gene sequences that contributed to the reduced data set shown in Figure 1B.
(0.01 MB TXT)

File S4 *Vmn1r67* gene sequences used to construct the gene tree for CODEML analysis.
(0.01 MB TXT)

File S5 Synonymous and nonsynonymous sites in *Vmn1r67*. Synonymous and nonsynonymous sites where apparent homoplasy was discovered when the species tree was used instead of the gene tree in CODEML analysis.
(0.00 MB TXT)

File S6 *Vmn1r67* amino acid sequences used in constructing Fig. 3.
(0.00 MB TXT)
